# Hemorrhage of the Mouth in an Infant 9 Days Old, Stomatitis Parasitica

**Published:** 1887-08

**Authors:** Rudolph Menger

**Affiliations:** San Antonio, Texas


DANIEL'S

### Texas Medical Journal.

Published Monthly.—Subscription $2.00 a Year.

Vol. 3. AUSTIN, AUGUST, 1887. No. 2.

### Original Articles.

CONTRIBUTED EXCLUSIVELY TO THIS JOURNAL.

*The Articles in this Department are accepted and published with the understanding that we are not responsible for, nor do we indorse the views and opinions of the writers by so doing.*

### HEMORRHAGE OF THE MOUTH IN A CHILD AGED 9 DAYS, AFFECTED WITH STOMATITIS PARASITICA—AUTOPSY AND MICROSCOPIC EXAMINATION.

*By Dr. Rudolph Menger, San Antonio, Texas.*

Read before West Texas Medical Association, San Antonio, Texas.
For Daniel's Texas Medical Journal.

AT THE last meeting of the West Texas Medical Association at San Antonio, a well attended and very interesting meeting was had. Besides my case on hemorrhage of the mouth in a child aged 9 days, a very interesting and perhaps unique case was related by Dr. F. Herff, in which he operated on a man for stone in the scrotum. The man had presented himself to Dr. Herff with a very large, tumor-like scrotum, of hard and nodulated appearance, and he had agreed upon to extirpate the entire scrotum. When the knife entered the tumor dartos, it came on a hard, solid calcarious mass, occupying the space of the cremaster, or rather, the entire cremaster was converted into a solid calcarious mass of nodulated form. This deposit was encapsulated, and so hard, the Doctor said, that it nearly broke his knife on reaching the stony tumor. The testacles were found to be entirely normal, and were, of course, not interfered with, after removing the entire stone, and also a number of large lymphatic glands in the inguinal canal.

R. MENGER.

In the following essay, I have taken the liberty to record, as near as possible, the details of a rare and interesting case, in which a child, aged only nine days, was affected with a very profuse hemorrhage of the mouth, and hemorrhagic extravasations in other organs. The hemorrhage from the mouth, which was a persistant oozing of blood, lasted over seventeen hours before death occurred.

It was on July 9th when I was called to attend the infant. I found it in a very precarious condition, and bleeding profusely from the mouth, especially from the tongue. There were large blood-clots in the mouth, and bleeding points along the rim of the gums and upper palate. The gums, cheeks, both sides of the tongue, and the palate, showed several granulated and rough surfaces, as if they were caused by some corroding substance, perhaps some acid or lye. The child had fever, and was much emaciated. After ordering ice applications to the mouth and abdomen, and a mucilaginous mixture containing ergot and catechu, I left the child, and saw it afterward, in company with Dr. Hertzberg, when the condition of the child was found no better. The blood had accumulated in quantities in the mouth, so that several large clots had to be removed with some difficulty. The abdomen was now very hot and highly tympanitic. After giving further instructions, I saw the child twice again, when, in the following morning, I was told that it had succumbed at one o'clock at night.

As the case proved to be of unusual interest, I made a post mortem examination the same morning, July 10, assisted by Dr. Favre, the result of which is as follows: Autopsy about nine hours after death. Body of child anæmic and emaciated; thorax weakly developed, showing the ribs elevated abnormally; abdomen much distended and tympanitic, with large and engorged veins running across the abdomen; numerous ecchymosis and petechiæ on body. The following is the general appearance of the mouth, pharynx, larynx, trachea, œsophagus, both lungs, heart, liver and intestines: The mucous lining of the lips, cheek, upper and lower gums, and palate, and sides of the tongue, was covered in different places with a mass of granular, curdy substance, of brownish color, protruding more or less,and firmly adherent to the mucous membrane. The tongue was covered with a thick, red-brown and bloody substance, of spongy appearance, covering nearly the entire surface of the tongue. This mass could be removed with a scalpel from the tongue, leaving quite an even surface on the tongue.

The œsophagus, which had been opened in its entire length, showed numerous nodulated protuberances, caused from parasitic deposits of the *oidium albicans*, else the same and also the pharynx were perfectly normal, without the least signs of bloody extravasation.

The opened larynx and trachea contained some streaks of coagulated blood, which, it appeared, had been aspirated from the mouth.

Both lungs were congested enormously, and of hemorrhagic appearance on the surface; heart largely developed, else apparently normal.

The stomach was distended and somewhat congested; the liver congested; bowels, especially ileum and lower colon, highly engorged, and of general hemorrhagic appearance; no bloody extravasation in abdominal cavity.

Being thus far highly interested in this case, I made a number of microscopic examinations from the different organs, after having them hardened. The first attempt made was to examine, as closely as possible, some of the remnants in the mouth, especially the large red-brown covering of the tongue, from which the main hemorrhage of the mouth seemed to have started. Glycerine mounts from this deposit showed two distinct substances, namely, a red-brown body, consisting mainly of epithelial elements impregnated with blood corpuscles, and a whitish body of granulated and amorphous appearance, consisting chiefly of elastic tissue, epithelial elements, and large quantities of a fungus, the *oidium albicans*, the filament and spores of which were seen projecting in different directions throughout the masses. Beside these bundles and filaments of fungi, there were swarms of bacilli distributed on the outside of the epithelial elements among a large number of blood corpuscles and detritus. There were no pus corpuscles present.

The blood corpuscles, and most of the other elements, were of a granulated character, somewhat retracted, and showed better after a day's masceration in glycerine under the cover-glass.

Tqe next effort made, was to determine if the colored surface of the tongue, which had been covered with the above red-brown substance, was caused by hemorrhagic infiltration. Several slides from this part of the tongue, and also from deeper parts, showed that the muscular fibres were of yellowish-red color, and impregnated with remnants of extravasated blood, the adjoining tongue-tissues being normal.

Particles of the granular substance on the mucous lining of the palate, and from the edge of the tongue, were now examined, and the result was that they showed nearly the same form elements as those of the red-brown deposits on the tongue; that is: epithelial elements and elastic tissues impregnated with the filaments and spores of the *oidium albicans*. Particles of deposits removed from the trachea also showed that they consisted of granulated corpuscles and epithelial elements.

The lung tissue showed throughout highly engorged vessels, with some blood extravasation in the inter-celular spaces, and in a few alveoli; no tubercular deposits.

Now, from the above, it is evident that the child was affected with stomatitis parasitica, or thrush, which, although on account of the hemorrhagic condition of the mouth, could not be distinctly diagnosed ante mortem. Thrush is, as we know, and according to the statement of Cohen in Pepper's System of Medicine, a minute granular substance, adherent to the tissues which often bleed when the substance is removed forcibly. In children much reduced by inanition or severe disease, much of the deposit soon coalesces into a membrane form product, grayish or yellowish from rarefaction of the air, or even brownish from admixture of blood. The characteristic masses under microscopic inspection are seen to be composed of the filaments and spores of a confervoid parasitic plant, the *oidium albicans*, enclosing altered epithelia in various conditions. This parasitic growth does not become developed upon healthy mucous membrane with normal secretory products. Acidity of the fluids and exuberance of epithelium are the requisites for its production, whatsoever be the cause. The acidity of the fluids irritates the mucous membrane, induces irritation and abnormal proliferation of epithelium, upon which the spores of the cryptogam then germinate. The disease is more frequent in the first two weeks of life than later.

Now, the main point of interest in our case is, and the question arises: "What produced the hemorrhage in the mouth?" Could it have been caused by the fungoid growths or the bacilli, as no such case, as far as I am aware, has ever been recorded in such delicate infancy; or can it be said with any certainty that it was produced through some corroding substance, as the general appearance of the mouth, and especially the microscopic examination would undoubtedly have been different; and what produced the congested condition in nearly all inner organs? Was it caused from the high fever, and consequently the increased blood pressure? or was there a general hemorrhagic diathesis or hæmophilia? The latter seems to me to be more plausible, as, according to the mother's statement, she had rubbed the child's tongue with a cloth saturated in salvia tea in order to remove the thrush, after which a bleeding was noticed, and it is well possible that from the plethoric condition of the infant, a rupture of the delicate vessels on the denuded surface of the tongue was caused, with uncontrolable hemorrhagic extravasations in the superficial and deeper tissues of the tongue. As far as the family history is concerned, and if it can be trusted, there is no hereditary, especially no hemorrhagic, tendency either in the parents or grand parents, although the mother of the child has been subject to epistaxis of late. The family consists of eight living children, all apparently healthy. One died when about two years old, from pneumonia. The mother stated, also, that the child had some bloody discharge from the bowels the day before I was called, and this is readily corroborated by the post mortem examination.

Dr. William Osler, in an elaborate treatise on hæmophilia, says: "The hemorrhagic diathesis may develop in children or members of a healthy family and prove fatal; and the question in such cases always comes up, are they instances of hæmophilia? There seems to be a desire to limit this term to cases of an hereditary nature only; but when a child shows a marked tendency to multiple hemorrhages, spontaneous or traumatic, which tendency persists and is not merely transitory, and particularly if there are joint troubles. I think that under these circumstances we have a genuine case of hæmophilia; and such a child, if he (it is more likely to be a male)

survives and marries, may be the founder of a bleeder family." In the history of the bleeder families we frequently come back to the origin in a person born by a healthy stock in which there have been no hemorrhagic tendencies. On the other hand, single, severe, uncontrolable hemorrhages in children or adults are not to be ranked as hæmophilia, unless there have been other features pointing to the existence of the diathesis.

In looking over the works of Virchow, Wagner and Uhle, Bock's Pathology, Pepper's System of Medicine, Henoch's work on diseases of children, Schmidt's Jahrbücher der Medicin, and a large number of journals, I find only one account described, in Henoch's work, in which a child, aged four years, died from hemorrhage of the mouth and nose, and this child had been afflicted with tuberculosis, the post mortem examination revealing abundant miliary tubercular deposits in both lungs, on the pleura, in the spleen, both kidneys, liver and lymphatic glands. Henoch says that he himself has never met with a similar case, neither before nor after, nor could his friend, Jacubusch, find a similar case in the literature.

From these accounts it will be seen that the above case of hemorrhage from the mouth, in a nine days old child, is a most rare, if not unique, case on record; and I therefore desire to state that I feel myself amply repaid for the time and trouble I have taken in recording the same.

